# Disseminating Truth and Doing Good “A La Mode”

**Published:** 1886-01

**Authors:** 


### DISSEMINATING TRUTH AND DOING GOOD "A LA MODE."

The following readable article is taken from *Daniel's Texas Medical Journal* for October, 1885:

The manner of doing good, and of disseminating truth, varies according to the notion of the disseminator, and the kind of truth to be disseminated, as well as with the kind of good to be done, and the class of individuals who are to be the recipient. Thus, ecclesiastics adopt the preaching of the gospel and the distribution of Bibles as a method of doing good from their standpoint. The modern philanthropist, with an eye to the more substantial nature of the good, endows a college or founds a hospital. An idea of doing good, once greatly in vogue, is admirably portrayed by Dickens, in the character of Mrs. Jellaby (Bleak House), whose whole soul was in sending red flannel shirts and Bibles to Borrioboola-gah, to the young savages, while her own young savages were crying for bread and falling over the bannisters. An individual philanthropist, a gambler, perhaps, may have an erroneous impression that he is doing good when he slips a five dollar bill into the hand of a shivering, ill-clad female mendicant at the street corner, or sends a barrel of flour "all unbeknown'st," or a load of coal to Dick Dooley's widow, left forlorn, with six little savages, by the falling of the walls of a burning building on

Dick, while he, poor fool, was giving expression to *his* crude idea of good by trying to rescue a child from the fourth story. There are, we say, many ways of doing good, and of disseminating truth; but commend us to the modern, of which we have an illustrated edition just to hand, in the shape of a pamphlet, a reprint with a *photograph*, and a hospital and a college on it.

In this age of progress and civilization there are those who think that if they can do good to an individual, a class, or a community, and at the same time *benefit themselves*, they are doing a double good, and that Heaven's recording clerk should score two points to their credit. This was the idea, no doubt, of the Pharisee, who thought he should *have credit* for giving alms. Why not? If one can do good to one's neighbor and to one's self at the same time, *why not?*

They say great minds run in the same channel. Undoubtedly Professor Windy Bowel, M. D., thought as we do, when he attached his photograph to a reprint of a learned article he had written (full of truths, no doubt), and with another picture of his hospital, and the following extraordinary address to "Dear Doctor," sent them to the medical profession. The diagnosis of this hermaphrodite pamphlet would have been obscure but for the address:

"DEAR DOCTOR—It is manifestly the duty of every upright citizen to disseminate truth and to do good. Upon this principle, and in strict accord with *the* ethics, I send you this pamphlet. I do not assume to be wise above others, but nevertheless I have a hospital, any number of boarding houses at my command, and all the latest and most approved facilities for every phase of gynecological practice [which you fellows haven't got]; therefore you must send such cases as fall into your hands on to *me!* * * * My photograph is designed as a response to the request of ever so many persons whom I shall never meet in this life, and who have expressed a desire to see my face." [! ! !]

Mind you, this pamphlet was not sent to the Heathen, whose soul is pictured as starving for "the truth," and on whose dark mind the light of gynecology never shone; nor yet to the suffering females, with lacerated perineii or cervices, whose sad souls are also athirst, and crying out in anguish for gynecological light. Oh, no; *they* could not reciprocate; that would be only a single good, and the recording clerk couldn't see his way clear to a double score for the Professor; but they are sent to the doctors,—to those who have not "approved facilities" and a hospital, and who are famishing for one brief, soul-snatching glimpse of that benevolent phiz.

We confess we *were* sad to think *we* might never meet him in this life; but now, having been permitted to drink (second hand) at the fountain of truth and goodness, so to speak, to gaze on the broad expanse of that *cheek*, to metaphorically kiss the hem of the great man's garment, we have arisen, like Sally in the song, and wiped our eye out with our frock, and are happy. We feel better. Our friends all feel better, too, notwithstanding our recent attack of dengue, since they, like ourselves, have seen that ethical pamphlet, pregnant to bursting with truths, and only held down by *the* photo in front, and *the* hospital in rear, like soldiers, fore and aft, guarding a convoy of precious treasure.

Ah, but our souls go out in sympathy with the unfortunate ones who may never hope to meet this modern Father Grimes, that good old man, who, although he has "no malice in his heart, no ruffle in his shirt," has better far than these—he has the "most approved appliances," and a hospital, and scores *and* scores of boarding houses; nor yet to behold even the shadow of that royal countenance! May we not hope that within that distant Aiden (too commonly called the sweet subsequently), they may clasp this rare and r-idiculous personage who so taketh the name of ethics in vain?

The medical profession should get up a testimonial for him, even if it be but an humble leather medal.

### ITEMS.

DR. E. H. RICHARDSON, of Cedartown, was in Atlanta a few weeks ago.

DR. A. P. THORNTON, a prominent physician of Thornton, Texas, was in Atlanta a few days since, and gave us a pleasant call.

AFTER January the Detroit *Lancet* will be known as the American *Lancet*. It will remain under the editorial management of Dr. Conner.

DR. J. W. STONE, the popular representative of Parke, Davis & Co., of Detroit, was in our city a few weeks ago, and gave THE JOURNAL a pleasant call.

DR. E. N. SHAW, a prominent physician of Cameron, Texas, was married to Miss Kate Gaston, daughter of Dr. J. Mc F. Gaston, of this city, December 12th.

There were twenty-five deaths from hydrophobia in London last year from January 1st to December 12th, the average number for the past ten years, 1875–1884, being six.

THE Newark children, who sailed from New York some weeks ago to be treated by M. Pasteur for hydrophobia, have arrived safely, and are now (December 24), under treatment.

DR. W. H. MARRIETT, representing Lee Bros. & Co., of Philadelphia, sold fifty-seven sets of Pepper's System of Practical Medicine and Surgery in Atlanta, amounting to over $1,700.00

WE had a pleasant call a few days since from Dr. M. Salm, of Austin, Texas. Dr. Salm was at one time a resident of Atlanta.

AT the last meeting of the New York Academy of Medicine, the following physicians were nominated for officers: For President,

Drs. Henry Noyes and Wm. M. Draper; Vice-Presidents, Drs. G. L. Peabody and A. M. Jacobus; Recording Secretary, Dr. Roosa; Treasurer, Dr. Bryant, and Dr. A. R. Robinson, Corresponding Secretary.

TREATMENT OF CICATRICES OF THE FACE.—In the treatment of cicatrices of the face, particularly of the lower jaw, all unsightly scars may be avoided by using a dressing of perchloride of iron, ʒi; collodion, ʒij. Let the cicatrix be cut clear off and the dressing applied every day, and a barely perceptible line will be the result.—*Dr. Genese, Maryland Medical Journal, September 12, 1885.*

A COMPLIMENT.—Some years ago there was at the University here a young fellow, from the neighborhood of Rome, by the name of Alexander. Bright, a good speaker, and a man of ready parts, he was quite an orator in the literary societies. That was J. Hooper Alexander, now of Atlanta.—*Athens Chronicle.*

Our readers will remember the article on "Crowner's Quest Law," written by this young man, which appeared in THE JOURNAL several months ago, and which was republished by several of our exchanges, among the number that sterling journal, the *Birmingham Medical Review*, of England.

PRECAUTIONS AGAINST HYDROPHOBIA.—A hydrophobia association has been formed in London, with a representative provisional committee, the secretary being Mr. George Murray, 64 Jermyn street, S. W. This society has been formed, as the prospectus tells us, with a view to check the great increase in the number of deaths from hydrophobia which has lately taken place. 1. To enforce existing laws as to the capture and destruction of stray and ownerless dogs. 2. To move Parliament to further legislation in reference to dogs by an increase of the dog tax, and by making it compulsory to have the name and address of the owner on every dog's collar, and a ticket denoting that the tax has been paid. 3. To obtain all possible information on the cause, symptoms and treatment of the disease, and to make the conclusions arrived at known to the public. 4. To take all such steps as may be considered necessary to further the objects of the association.—*British Medical Journal, December 12.*

HE NEVER MADE A MISTAKE.—Young Doctor: "Indeed, madam, I know all about it. I saw just what ailed this woman as soon as I came into the room. There is a young man up street who is sick with the same trouble, and I am attending him. Give the medicine as directed and I will call around in the morning."

Not long after the young man departed, the pains returned, and a messenger was sent for the old doctor, who, it was found, had just returned from a far-out call, and he gave the necessary attention to the case.

The young doctor returned next morning and congratulated himself on the happy appearance of his patient, saying: "You see, I never make any mistakes." Whereupon the nurse turned down the covering, and showed him the little one lying in the mother's arms, and asked quizzically: "Doctor, how is the young man up street this morning?"—*Ex.*

VERRUGA: A PERUVIAN FEVER.—The Lima Academy of Medicine has determined to make as many inquiries and observations as possible, in order to elucidate the natural history of a terrible Peruvian disease, known as verruga, which is thought to be identical with the fatal "Oroya fever," which many years ago carried off many of the work-people employed on the railway across the Andes. Quite recently, a medical student, named Daniel A. Carrion, with ill-advised zeal for the advancement of science, inocuated himself with verruga, in the Dos de Mayo Hospital, hoping to be able to gather some new facts about the disease, for the special purpose of enriching a dissertation he was writing on it for his approaching graduation. Some facts were certainly obtained, but, sad to say, at the cost of the life of the zealous experimentalist, who died thirty-eight days after inoculation, his symptoms being adynamic pyrexia, general dermatitis and a morbid change in the blood, resembling leucocythemia. The period of incubation was twenty-three days. His self-inoculation was quite unauthorized by his teachers, who naturally deeply deplore so sad a termination to the career of a most promising student.—*British Medical Journal, December 12.*

PROFESSOR VIRCHOW has recently asserted that "no specialty can really flourish which cuts itself entirely off from the great body of the science."

TYPHOID fever is said to be unusually prevalent in certain portions of Brooklyn, the special cause for which does not seem to have been distinctly determined.

DR. SQUIBBS suggests as a means of disguising the taste of bitter and nauseous salines a mouthful of ice-water just before and just after the dose, the saline itself to be taken in a wineglassful of iced water between them.

AT the annual meeting of the Atlanta Society of Medicine, held December 1, 1885, Dr. J. Donald Wilson was elected President; Dr. N. O. Harris, Vice-President; Dr. Virgil O. Hardon, Secretary; Dr. W. B. Parks, Treasurer; Dr. W. S. Elkin, Librarian and Corresponding Secretary; Dr. C. C. Green, Censor; Drs. F. W. McRae, Henry, Wile and E. Van Goidtsnoven, Reporters.

THE NUMBER OF DOCTORS IN ATLANTA.—There are two hundred and sixty-seven names of physicians on the register in the clerk's office of this county. The law requires that every one registering shall make affidavit as to the authority for practicing medicine. Among authorities given, we find the following: One from the "State Board Eclectic Physicians," one "Board Medical Examiners of Georgia," one "Botanic Medical Board of Georgia," one "Board of Physicians of Georgia," one "from Dr. Samuel Thompson," one "Georgia Eclectic Board," and one "Georgia Medical Board."

A NEW ETIOLOGICAL FACTOR.—Our Doctor Merriman denies that strabismus is due to a "*micro-cock-eye.*" He says that a number of recent cases, at least, have been produced by frantic efforts to "see the point" of some of *The Age's* jokes.—*Daniel's Texas Medical Journal.*

TRICHINOSIS.—The New York correspondent of the *Medical News*, December 12: The occurrence of eight cases of trichinosis among the members of a German family, who had previously enjoyed themselves at a birthday feast in which raw ham figured, is reported. There have been but four cases of the disease in this city since its discovery, and it would seem that imported ham was responsible in every instance.

A MISTAKE IN DIAGNOSIS.—A young man fresh from college, whence he came with honors and medals, was sent by his father, a practitioner of fifty years' standing, to attend a woman in labor. On making a digital examination, he found the os uteri undilated. After waiting an hour, there being no improvement, he applied belladonna ointment, and endeavored to make forcible dilatation. At the end of another hour, there was still no dilatation ; and, being alarmed, he went to his father for assistance; but before they returned the child was born. On examination, the father found that the child's anus was red and patulous, and was liberally besmeared with belladonna ointment. The young practitioner had met with a breech-presentation, and had mistaken the child's anus for an undilated os uteri.—*Northwestern Lancet.*

BROMIDIA.—Dr. Thomas Stretch Dowse, of London, in a letter to the manufacturers of this preparation, says: "I am glad to speak in praise of your preparation named bromidia. As it is a known compound in quantity and quality, its virtue should be known, and not even the most fastidious of physicians can refuse to prescribe it. I have found it a most valuable hypnotic and sedative, and it unquestionably controls the circulation of the brain generally, and the nervous centres and gray matter in particular. In muscular spasm and in epilepsy its effect is very marked."

TRIBUTE OF RESPECT.—At a meeting of the Macon Medical Society on the 16th instant, the following resolutions were unanimously adopted:

Death, at all times an unwelcome visitor, has again invaded our circle and removed from us a comrade and fellow-worker, Dr. E. Fitzgerald, one of the original members of this society, and an honest and zealous worker for its prosperity and advancement. We, the surviving members, desire to give expression of our sorrow at the death of one who, by his noble traits of character and exalted professional attainments, won for himself the position of honored friend and brother. Therefore be it

*Resolved*, That in his death this society has been deprived of one of its most useful and gifted members. The community has lost an honorable citizen and chivalrous gentleman, and his family a devoted husband and father.

*Resolved*, That we tender to his family our heartfelt sympathy in this their hour of heavy sorrow and deep bereavement.

*Resolved*, That these resolutions be inscribed upon the minutes of our society, and that a copy be sent to his family and one furnished the city papers for publication.

WILLIAM F. HOLT,
CHARLES H. HALL,
D. W. HAMMOND,
Committee.

R. O. COTTER, Sec'y.
Macon, Ga., December 17, 1885.

A CORRECTION.—On page 671 of this JOURNAL, fifth line, injestion should be read for injection.

*Daniel's Texas Medical Journal* for December is an exceptionally fine number.

DEATH AFTER PHIMOSIS OPERATION.—Dr. S. B. Bond, Chief of Clinic to the Professor of Surgery, University of Maryland, reports a case (*Maryland Medical Journal, Dec. 12th*) of gangrene following operation for phimosis, which resulted in death on the fourth day after the operation.

A CASE OF VARIOLOID.—From the Savannah *Morning News*, of December 23, we learn that a case of varioloid has occurred in that city. The patient, a man, came from New York by steamer about a week ago. Every precaution has been taken to prevent the spread of the disease.

NEW YORKER MEDIZINISCHE PRESSE.—The first number of this newly organized medical monthly comes to our notice. It is printed in the German language, and claims to be the organ of the German-American physicians. The necessity for such a journal is obvious, and it supplies a want long felt by our German colleagues. The journal is creditably gotten up, and its contributors are men of recognized ability.

This issue contains several original articles by well known specialists, a salutatory editorial, and the latest medical news, together with gleanings from leading European journals. The *Medizinische Presse* begins well and deserves to succeed.

A CURIOUS MONSTROSITY.—A child was born recently in Naples having a fifth limb, corresponding to the right lower extremity, going from the region of the false ribs on the left side near the sternum. There were well-developed male genital organs in the normal situation, and another set, complete in every respect, except that the testicles seemed to be wanting, on the inner side of the thigh of the supernumerary limb. The patellæ were absent, and a fold of skin prevented full extension of the leg. The skin and muscles of the abdominal wall were wanting in the umbilical region, so that the intestinal movements could be distinctly seen beneath the peritoneal covering. The pavilion of the left ear was also double. At last accounts this child was alive and in good health.—*New York Medical Record, December 19*.

THE OPIUM HABIT IN TEMPERANCE TOWNS.—Dr. R. C. Downey, of Caldwell, O., writes us that he has been much astonished at the large sales of opium and morphine in small towns. He lives in a place of 1,500 inhabitants. He writes: "I called at one of our two drug stores for a drachm of morphine. The druggist said he was out of it. I asked, 'What has become of the large amount you had only a few days ago.' He replied that his regular customers had taken it all. He declined to tell me who they were, but said he had one customer who bought it by the ounce." Dr. Downey thinks that he has obtained facts which show that opium-eating is more common in temperance towns and communities than in those in which the liberal use of alcoholie drinks is allowed.—*New York Medical Record, December 19*.

A NEW TEST FOR HUMAN MILK.—Dr. Helot (Lyon *Medical*, October 18th) has discovered a method at once simple and practical to test the quality of woman's milk. While, of course, chemical analyses are reliable, yet they are so long and difficult that M. Tarnier has recommended physicians to accustom themselves to recognizing the richness of milk by the sight. Over this method, that of M. Helot has the great advantage of precision. It consists of a comparison by means of a dropper of the number of drops in a volume of distilled water at 15° with that in an equal volume of the milk. Good milk, that which will cause a gain of 25 gm. per day in the weight of the child, gives a proportion of 35 drops to 30 of distilled water. The number of drops may vary, increasing to 36, 37 or 38. The milk is then of superior quality. If, on the contrary, one gets only 33 drops, or less, he should mistrust the milk. The Pravaz syringe allows of the accurate accomplishment of this examination, it only being necessary to remember the ratio of 5 to 6 between water and good milk. The test should be applied to each breast in the middle of the nursing.—*Boston Medical and Surgical Journal, December 17.*

A DOCTOR FINED FOR REFUSING TO TESTIFY IN COURT.—The *Medical Record*, December 5, 1885, notes that a daily paper announces that Dr. J. A. Milne, of Oswego, New York, was, on November 25, fined $250, with the alternative of six months' imprisonment, for contempt of court in refusing to testify in a criminal case. Dr. Milne pleaded, as a defense for his refusal, that his testimony would involve the disclosure of professional secrets. The law in New York, as well as in Arkansas, California, Indiana, Michigan, Iowa, Missouri, Minnesota, Montana, Ohio and Wisconsin, is that communications between physicians and patients which may relate to the history of a transaction, in which a wound has been received, or a particular disease communicated, whenever essential to the treatment of a patient's case, are privileged. In New York it is even expressly enacted that the physician shall not disclose any information which he may have acquired in attending any patient in a professional character, and which information was necessary to enable him to treat the patient.

PASTEUR'S WORK IN HYDROPHOBIA.—The London correspondent of the *Journal of the American Medical Association*, Dec. 12th, says: The world will owe a lasting debt of gratitude to M. Pasteur if he has really discovered how to cure that most horrible of maladies, hydrophobia. He believes he has proved it to be curable, and, in support of his assertion, he has communicated to the French Academy of Sciences the results of his experiments and researches relating to rabies in the human subject. M. Pasteur had, in the course of the summer, an opportunity of testing the value of his method on a boy who had been recently bitten by a dog unquestionably proved to have been mad at the time of the accident. The boy had been brought from Alsace by his parents to be placed under the eminent Professor's care. He was examined by M. Pasteur and two distinguished medical men; fourteen bites were found about his body, and the symptoms of the dreaded disease were already evident in the patient. In the space of ten days the child was inoculated no fewer than thirteen times on the same system as M. Pasteur has successfully tried with dogs. This was three months ago, and until now nothing has occurred in the condition of the patient to shake the operator's faith in his method. The boy continues in excellent health, and M. Pasteur believes that he is not only safe from hydrophobia caused by the bites of the dog, but also from any bad effects resulting from the animal virus introduced into his system to insure his safety from rabies.

Four little children were sent to Pasteur from Newark, N. J., early in December last, for inoculation with the virus of hydrophobia, they having been bitten by mad dogs in Newark several days before.

THE MAN WITH INTERESTING CASES.—New York must be infected with a class of doctors that the South is not free from, judging from the following editorial taken from the *Medical Record* of December 19th : The young medical man who launches his (largely Peruvian) bark upon the unhealthful seas of medical practice has many discomforts, obstacles and dangers to encounter. Not the least among the minor ills—to drop our meritorious but ancient simile—is that of the man who always has a case to describe.

Some distinguished medical philosopher has remarked that he would rather be knocked on the head at once than be stopped on the street and told about a case. This gentleman evidently had had some painful experiences which perhaps warped his judgment a little; for, after all, to be knocked on the head is not only disagreeable, but serious, while the man with the case is a depressive force rather than a destructive one.

Every one knows him, however, this man of many cases; he meets one at the street corner, at societies, he drops in at the office. He greets his victims with a pleasant "Good-day-how-is-practice-last-night-I-was-called-to-see-a-" etc., manner, and in a moment he is in the midst of bedside details of anguishing minuteness. One listens in a dazed way, catching now and then something about "injections," "temperature," "bowels," "bladder," and similar expressions; while perhaps the speaker ends temporarily with an explanation of why he called some other gentleman in consultation.

The case-fiend penetrates every stratum of society. To tell the story of his experiences with unhealthiness is to him a kind of erotic joy. His cook, his wife, his druggist, the poor, the rich, the sick, the well, all get a share of his case-book. He drops into a case-recital as easily as Joseffy into a sonata, or a *premiere danseuse* into a pirouette; his intellectual life is in the verbal revival of his pathological experiences; of these he is a word-painter, pre-Raphaelitic in detail, persistent in purpose, and exhaustless in resources.

He is seen at societies, and here he acts as a dispersive force of singular potency upon members, and respectable gentlemen of our medical profession of high standing and large practice will steal on tip-toe out of side-doors like thieves in the night.

But it is, after all, in his private relations that the man of cases is at his best, and the question of what shall be done with him is a medico-social problem of deep interest.

For our part, we urge upon these gentlemen a prompt reform. We would assure them that in many departments of medical knowledge, clinical recitals can add nothing, and that in some directions the end of clinical research has been nearly reached. This should make these more than Roman viaducts of bed-side studies very careful. They should reserve these exercises of their memories, and cultivate somewhat more their logical faculty and the powers of interpreting the meaning of things. This bedside tattle of "cases" is too often the same kind of stuff as the gossip of a ladies' dressing-room.

FRIEDRICHSHALL MINERAL WATER.—Some further improvements are in course in the method of caption of this old-established and favorite mineral aperient water. These, by preventing the access of sweet water, and avoiding the dilution of the mineral water, will considerably increase its mineral strength and efficacy. Such improvement will doubtless be of considerable value, as it will enable the water to be used in smaller bulk, and, therefore, more conveniently than heretofore, and thus its efficacy in practice will be increased. The testimony of Bamberger, Frerichs and Seegen, and other foreign as well as eminent British authorities, has for many years concurred in indicating Friedrichshall Water as an aperient which, for habitual use, is unrivalled. Its richness in chlorides gives it especial alterative value, and there is a great body of physiological evidence, collected by Mosler and Mering, as well as of clinical evidence from the hands of Sir Henry Thompson and others in this country, indicating the especial value of Friedrichshall in preventing the formation of uric acid, and establishing the fact that, unlike mineral aperients, its dose may be progressively diminished during continuous use. The improvements in the method of capturing and bottling the water, so as to free it from dilution, will no doubt add to its long-established popularity.—*British Medical Journal.*

A CORPSE IS NOT PROPERTY.—The New York *Herald* says that the Supreme Court of South Carolina has recently rendered an elaborate opinion on a question which appears to have been decided for the first time in that case. It was an action brought by an administrator against a railroad company to recover damages for the mutilation of a corpse, caused by the negligent running of a train over it. The referee found for the plaintiff, and fixed the damages at ten thousand dollars. The case went to the Supreme Court on the question whether "there is such property or interest in the dead body of a human being as to sustain an action for its willful or negligent mutilation, and, if so, whether the right of action belongs to the administrator of the deceased."

The Court decides that, while the next of kin have recognized rights touching the dead, there is no such property in a corpse as is claimed in this case. It says: "While the remains of the dead should be tenderly protected, their interment carefully guarded, the mutilation of their bodies and the disturbance of their sepulchre severely punished, and while all laws necessary to that end should be passed and strictly enforced, yet, even for this purpose, to make such remains the absolute property of any one in the sense of objective appropriation would be abhorrent to every impulse and feeling of our nature."

But this view, the Court proceeded to explain, does not apply to the clothes in which the body was clad, and the watch on it. These were articles of personal property to which the administrator had a legal claim, and as they were destroyed he was entitled to maintain an action for their value.—*Boston Medical and Surgical Journal.*

EPHEMERIS DISCONTINUED.—Dr. Squibb announces the discontinuance of the interesting little series of pamphlets which, for the past few years, he has issued under the name of "An Ephemeris of Materia Medica, Pharmacy, Therapeutics, and Collateral Information."

METEOROLOGICAL SUMMARY FOR THIS STATION FOR MONTH OF NOVEMBER, 1885.

| | |
|---|---|
| Highest Temperature, November 6th, | 73.0 |
| Lowest Temperature, November 26th, | 29.2 |
| Greatest Daily Range of Temperature, November 10th | 26.8 |
| Least Daily Range of Temperature, November 28 | 5.8 |
| Mean Daily Range of Temperature, | 16.0 |
| Mean Daily Dew-point, | 59.4 |
| Mean Daily Relative Humidity, | 69.9 |
| Prevailing Direction of Wind, | N. W. |
| Total Movement of Wind, | 7,365 Miles. |
| Highest Velocity of Wind and Direction, | 26 Miles, N. W. |
| Number of Foggy Days, | None. |
| Number of Clea Days, | 11 |
| Number of Fair Days, | 14 |
| Number of Cloudy Days, | 5 |
| Number of Days on which Rain or Snow fell, | 12 |

